# Molecular Markers of Ovarian Germ Cells of Banana Prawn (*Fenneropenaeus merguiensis*)

**DOI:** 10.3390/cimb45070360

**Published:** 2023-07-07

**Authors:** Tatiyavadee Sengseng, Tomoyuki Okutsu, Anida Songnui, Jaruwan Boonchuay, Chanida Sakunrang, Monwadee Wonglapsuwan

**Affiliations:** 1 Division of Biological Science, Faculty of Science, Prince of Songkla University, Hatyai, Songkhla 90110, Thailand; 2 Japan International Research Center for Agricultural Sciences, Tsukuba 305-8686, Ibaraki, Japan; 3 Trang Coastal Fisheries Research and Development Center, Department of Fisheries, Trang 92150, Thailand; 4 Center for Genomics and Bioinformatics Research, Faculty of Science, Prince of Songkla University, Hatyai, Songkhla 90110, Thailand

**Keywords:** ovarian germ cell-specific genes, molecular marker, oogenesis, in situ hybridization, banana prawn, *Fenneropenaeus merguiensis*

## Abstract

The banana prawn (*Fenneropenaeus merguiensis*) is a valuable prawn in the worldwide market. However, cultivation of this species is limited owing to the difficulty in culture management and limited knowledge of reproduction. Therefore, we studied the gene expression and molecular mechanisms involved in oogenesis for elucidating ovarian germ cell development in banana prawns. The tissue-specific distribution of certain genes identified from previous transcriptome data showed that *FmCyclinB*, *FmNanos*, and nuclear autoantigenic sperm protein (*FmNASP*) were only expressed in gonads. The in situ hybridization (ISH) of these three genes showed different expression patterns throughout oogenesis. *FmCyclinB* was highly expressed in pre-vitellogenic oocytes. *FmNanos* was expressed at almost the same level during oogenesis but showed the most expression in late pre-vitellogenic stages. Based on the highest expression of *FmCyclinB* and *FmNanos* in mid pre-vitellogenic and late pre-vitellogenic oocytes, respectively, we suggested that FmNanos may suppress FmCyclinB expression before initiation of vitellogenesis. Meanwhile, *FmNASP* expression was detected only in pre-vitellogenesis. Moreover, quantitative real-time polymerase chain reaction (qRT-PCR) analysis of *FmNASP* expression was supported by F*mNASP* ISH analysis based on high expression of *FmNASP* in sub-adult ovaries, which contain most of pre-vitellogenic oocytes. In this study, we found three reliable ovarian markers for banana prawns and also found a dynamic change of molecular mechanism during the sub-stage of pre-vitellogenesis. We determined the expression levels of these genes involved in oogenesis. Our findings provide information for further studies on banana prawn reproduction which may assist in their cultivation.

## 1. Introduction

The aquaculture sector and fisheries support the livelihood of people worldwide. Shrimps and prawns represent one of the most important commodities traded, accounting for 16% of the total value of internationally traded fish products in 2019 and in 2020, continuing the trended with recent years at 16.4% at a lower growth rate (3.3% in 2018–2019 and 2.6% in 2019–2020) (FAO 2023) [1]. However, the aquaculture of the shrimp and prawn group mainly includes cultivation of Pacific white shrimp (*Penaeus vannamei*) and black tiger shrimp (*Peaeus. monodon*) alone. Most species in this group are captured in fisheries. The banana prawn (*Fenneropenaeus merguiensis*) is one of the most economically important species globally. However, the banana prawns sold in markets are mainly sourced from the wild, owing to limited success in farming based on the high mortality of premature prawns and difficulty in culture management. Moreover, knowledge on banana prawn reproduction is still limited when compared to that of other shrimps or prawns, such as the black tiger shrimp, pacific white shrimp, and giant freshwater prawn [1,2]. Therefore, to improve the aquaculture of banana prawns, the development and mechanism of reproduction and ovarian maturation in banana prawns should be elucidated.

The development of ovaries in shrimp is a complex process that involves the expression of various genes [3]. Recent studies have identified several significant sexual genes, such as *Wnt4*, *CFSH*, *Dmrt* gene family, *Sox* gene family, cell-cycle gene family, and *Fem-1*. These genes play crucial roles in the direct or indirect regulation of ovarian development [4]. However, further investigations are required to explore additional sexual factors, including sex-related genes and molecular pathways, in various crustacean species [5]. By identifying and analyzing these sex-related genes involved in the regulation of ovarian development, valuable insights can be gained, potentially leading to the development of novel techniques for manipulating ovary development [6]. In the ovarian development of shrimp, members of the *Cdk* gene family are involved in the regulation of oocyte maturation and ovulation. For example, Cyclin-dependent kinase 2 gene (*cdk2*) plays an important role in the proliferation of oocytes and the synthesis and transportation of exogenous yolk material [7]. The cyclin gene family is also involved in the ovarian development of shrimp. The expression of the cyclin A (*CycA*) gene in the Oriental river prawn was gradually increased during the embryonic stage and decreased significantly on the first day of the hatching stage. The *CycA* dsRNA could delay the ovarian development cycle of oriental river prawn [8]. β-thymosin (PmTmsb) and Rac-GTPase-activating protein 1 (PmRacgap1) were reported as negative effectors during ovarian development in giant tiger shrimp [9].

Recently, a transcriptomic data analysis of banana prawn ovaries (Ikuo Hirono, unpublished data) revealed various genes that may be related to germ cell development in the banana prawn, including cyclin A, cyclin B, cyclin E, Nanos, meiosis regulator and mRNA stability factor 1 (*MARF1*), and nuclear autoantigenic sperm protein (*NASP*). In black tiger shrimp, qRT-PCR studies have reported that cyclin A gene expression is higher in ovaries of brood stock than in juvenile prawns, and cyclin B gene expression is higher in stage IV ovaries than in stage I ovaries [10]. A pull-down assay analysis of the cyclin E gene in black tiger shrimp showed interaction with cyclin-dependent kinase 2, which is a key mediator that controls G1/S transition [11]. The *NANOS* gene in humans is required for maintenance of undifferentiated spermatogonia [12], and the knockdown of the *Nanos1* gene decreases the number of primordium germ cells in the gonads of *Xenopus* [13]. The *MARF1* gene is required for female meiotic progression in humans [14] and regulates Nanos protein expression for oocyte maturation in the fruit fly [15]. The *NASP* gene expression is higher in the ovaries than that in the testes in black tiger shrimp [16], and it is expressed in all stages of ovaries in white shrimp [17]. However, there are no reports on the expression or mechanisms of these genes in banana prawns.

The gene expression and molecular mechanisms involved in oogenesis are essential for elucidating the process of ovarian germ cell development to improve banana prawn aquaculture. In this study, we aimed to identify germ cell marker genes by screening genes of interest RT-PCR. Genes which were expressed in gonads alone were further analyzed via sequencing and studied for their expression pattern and localization in ovarian germ cells via ISH. The expression level of each gene was validated by qRT-PCR. This study provides data for understanding the molecular mechanism of ovarian germ cell development to improve banana prawn breeding.

## 2. Materials and Methods

### 2.1. Animals

Banana prawns were collected from Trang Coastal Fisheries Research and Development Center, Trang, Thailand. Prawns were cultured for a few days at an aquaculture building in Prince of Songkla University, Songkhla, Thailand, in re-circulating seawater (30 ppt salinity) at 28–30 °C, pH 7.4–8.5. Prawns were fed with commercial feed twice per day (Thai Luxe Enterprises PCL, Phetchaburi, Thailand). All experiments were performed according to the Guidelines for the Care and Use of Laboratory Animals, Prince of Songkla University (approval no. 51/2021).

### 2.2. Tissue-Specific Distribution of Gene Expression

Total RNA was extracted from the testis, ovary, vas deferens, intestine, brain, heart, hepatopancreas, muscle, thoracic ganglion, eyestalks, gills, and epidermis tissues of male and female banana prawns (*N* = 5), using TRIzol™ Reagent (Invitrogen, Waltham, MA, USA) according to the manufacturer’s protocol. The first-strand cDNA was synthesized using random primers and AMV reverse-transcriptase enzyme (Promega, Madison, WI, USA) according to the manufacturer’s protocol. The cDNA from each organ was amplified using various primers (Table 1) that were designed from banana prawn transcriptome sequence results (Ikou Hirono, unpublished data). A pair of beta-actin gene primers [18] were used as an internal control. The PCR reaction mix comprised 500 ng cDNA, 250 mM dNTPs, 1 pmol of each primer, 2.5 mM MgCl_2_, 1 × GoTaq^®^ Flexi buffer, and 0.25 units GoTaq Flexi DNA polymerase (Promega). Amplification was performed with SimpliAm™ Thermal Cycler (Applied Biosystems™, Waltham, MA, USA) at 94 °C for 5 min, followed by 35 cycles of annealing and elongation step. The temperature and time for each step of each primer are shown on Table 1. The final elongation step was at 72 °C for 7 min. The PCR products were loaded in a 1.5% agarose gel that was prepared using GelRed^®^ Nucleic Acid Gel Stain (Biotium, Fremont, CA, USA). The electrophoresis voltage was 100 V, using 0.5 × TAE buffer for 30 min. The gel pictures were captured.

### 2.3. Sequence Analysis

The full-length sequences of genes expressed in reproductive organs were verified by cloning into pGEM^®^-T easy vector (Promega). The cDNA was synthesized from ovarian RNA, using AMV transcriptase enzyme (Promega), as described in Section 2.2. Recombinant plasmids were sequenced using Sp6 and T7 primers. The sequences of the genes were analyzed for predicting functions. The amino acid sequence of each gene-coding protein was analyzed for determining phosphorylation sites and kinase motifs, using NetPhos 3.1 sever (DTU Health Tech, Lyngby, Denmark). Multiple sequence alignment was performed with sequences of other crustacean species, using the GenDoc program, and a phylogenetic tree was constructed using sequences of other organisms, using MEGA version 7.0 via the Neighbor-Joining method based on the Poisson model.

### 2.4. Cloning of Partial FmCyclinB, FmNanos, and FmNASP Genes

Total RNA was extracted from ovaries, using TRIzol™ Reagent (Invitrogen), and cDNA was synthesized using AMV transcriptase enzyme (Promega), as described in Section 2.2. The partial *FmCyclinB*, *FmNanos*, and *FmNASP* genes were amplified using banana prawn ovary cDNA. The PCR product of each gene was purified using QIAquick^®^ PCR & Gel Cleanup kit (Qiagen, Hilden, Germany). The purified cDNA fragments were cloned into pGEM^®^-T easy vector (Promega). Recombinant plasmids were sequenced using Sp6 and T7 primers.

### 2.5. In Situ Hybridization (ISH)

ISH was performed as described by [19,20], with minor adjustments. Briefly, each recombinant plasmid was transcribed into antisense and sense RNA probes, using a digoxigenin (DIG) RNA Labeling kit (SP6/T7) (Roche, Basel, Switzerland). For ISH, the immature, early previtellogenic, and mature ovaries of banana prawns were fixed in Bouin’s fixative at 4 °C for 7 h and then placed in 70% ethanol/RNase-free water for 24 h at 4 °C. Dehydration was performed in a series of ethanol solutions, followed by embedding samples into paraffin. Each paraffin tissue was cut into 5 µm serial sections. The paraffin sections were deparaffinized and hydrated in xylene–ethanol series and then treated with proteinase K (Invitrogen), acetylated, and hybridized with hybridization solution of 1 µg DIG-labeled antisense or sense RNA probe at 65 °C for 18 h. The sections were then washed in 2 × SSC/50% formamide at 65 °C for 30 min twice, in 1 × SSC/50% formamide at 65 °C for 20 min thrice, and in 0.5 × SSC/1 × Tris-buffered saline and Tween 20 (TBST)/25% formamide at 65 °C for 10 min once. Nonspecific binding probes were digested using 10 µg/mL RNase A/NTE buffer (500 mM NaCl, 10 mM Tris-HCl pH 8.0, and 1 mM EDTA) at 37 °C for 10 min; washed twice in NTE for 5 min; and then washed thrice in 0.5 × SSC/1 × TBST at 65 °C for 20 min, followed by three washes in 1 × TBST at 25 °C for 5 min. Nonspecific binding was blocked with blocking Reagent (Roche)/1 × TBST for 15 min, at room temperature, in a moist chamber. The sections were incubated with anti-DIG-AP Fab fragments (Roche) (diluted 1:500 in blocking/1 × TBST) at room temperature for 90 min. The sections were then washed thrice in 1 × TBST for 5 min, followed by once in NTMT solution (100 mM NaCl, 100 mM Tris-HCL pH 9.5, 50 mM MgCl_2_, and 0.1% Tween 20). The sections were detected in detection buffer (0.0035% nitroblue tetrazolium (Promega) and 0.0018% 5-bromo-4-chloro-3-iodolyl phosphate (Promega) in NTMT) at room temperature in the dark. After the reaction occurred, the slides were counterstained with Nuclear Fast Red (Abcam, Cambridge, UK) for 5 min. The sections were observed under a light microscope (Olympus-IX 70, Olympus, Tokyo, Japan).

### 2.6. Quantitative Reversed Transcript PCR (qRT-PCR)

The shrimp were divided into two groups, a sub-adult and adult group, based on body weight and gonadosomatic index (%GSI). The body weight and %GSI of sub-adult shrimp were 20.10 ± 3.60 g and 0.47 ± 0.16%, respectively, while the body weight and %GSI of adult shrimp were 33.22 ± 6.68 g and 0.98 ± 0.15%, respectively. The %GSI was calculated as follows: GSI (%) = gonad weight × 100/body weight. Total RNA was extracted from sub-adult and adult ovaries (*N* = 5), using TRIzol™ Reagent (Invitrogen), and cDNA was amplified using AMV transcriptase enzyme (Promega), as described in Section 2.2. Each 100 ng cDNA sample was amplified using primers listed in Table 2. Beta-actin gene (Actin-F and Actin-R) was used as a reference gene. The qRT-PCR reaction was performed using Luna^®^ Universal qPCR Master Mix Kit (New England Biolabs, Ipswich, MA, USA) and subjected to qRT-PCR analysis (CFX96™ Real-Time System, Bio-Rad, Hercules, CA, USA). Amplification was initiated at 94 °C for 5 min, followed by 39 cycles at annealing temperature and annealing time for each primer. A dissociation curve analysis was subsequently performed at 95 °C for 20 s, 55 °C for 20 s, and 95 °C for 20 s. The standard curve for quantifying all genes was prepared using serial dilutions of the linearized purified PCR products of each gene. The dynamic range of detection was determined by preparing 10-fold serial dilutions of all genes in the range of 1 × 10^7^ to 1 × 10^3^ copies. The copy number of each reacted product was calculated according to its molecular weight and then converted into the copy number based on Avogadro’s number. The ratio of the copy number of each gene relative to the copy number of the beta-actin gene in each sample was calculated. Each sample was performed in triplicate. The primers for real-time PCR are shown in Table 2.

### 2.7. Statistical Analysis

The mRNA expression level was compared statistically, using the Independent-Samples T Test of SPSS at a 95% confidence level (*p* < 0.05).

## 3. Results

### 3.1. Tissue-Specific Distribution of Genes

**Figure 1:**
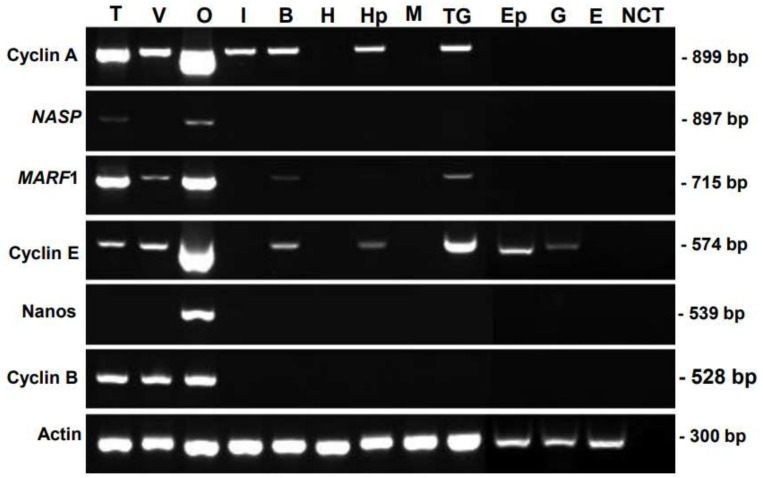
Tissue-specific distribution of candidate genes. Total RNA was isolated from T, V, O, I, B, H, Hp, M, Ep, G, and E. cDNA was amplified, and the full size of PCR product is in parenthesis: *Cyclin A* (899 bp), *NASP* (897 bp), *MARF1* (715 bp), *Cyclin E* (574 bp), *Nanos* (539 bp), and *Cyclin B* (528 bp). β-Actin gene (300 bp) was amplified as an internal control. A negative control without cDNA template is shown in lane NCT. T, testis; V, vas deferens; O, ovary; I, intestine; B, brain; H, heart; Hp, hepatopancreas; M, muscle; TG, thoracic ganglion; Ep, epidermis tissues; G, gills; E, eyestalks; *FmNASP*, banana-prawn-derived nuclear autoantigenic sperm protein; *MARF1*, meiosis regulator and mRNA stability factor 1.

The tissue specificity of candidate genes was examined via RT-PCR analysis, using various somatic organs and reproductive organs, which contain germ cells and/or gametes (Figure 1). The prawns used in this study were at the juvenile stage, which contained dominantly previtellogenic oocytes. *FmCyclinB* transcripts were detected in the testis, ovary, and vas deferens; *FmNanos* transcripts were detected in the ovary alone; and *FmNASP* transcripts were detected in the testis and ovary. In contrast, the transcripts of these genes were detected in none of the somatic tissues. These results indicated that *FmNASP*, *FmNanos*, and *FmCyclinB* were specific to reproductive organs. However, *FmCyclinA*, *FmMARF1*, and *FmCyclinE* expression was not specific to gonads alone; therefore, we selected *FmNASP*, *FmNanos*, and *FmCyclinB* for further study.

### 3.2. Sequence Analysis

The full-length cDNA of *FmCyclinB* (1486 bp), *FmNanos* (2700 bp), and *FmNASP* (2420 bp) (accession numbers OP156936, OP296393, and OP156937, respectively) was obtained from ovarian transcriptomic data of banana prawns (Ikuo Hirono, unpublished data). The putative amino acid sequences were predicted for phosphorylation site and sequence motif. The proteins contained various predicted phosphorylation sites and kinase motifs (FmCyclinB (Supplementary Figure S1a), FmNanos (Figure S1b), and FmNASP (Supplementary Figure S1c)). The number of predicted phosphorylation residues and type of predicted kinase motif are presented in Table 3. Kinase motifs such as PKA, PKC, CKII, ATM, DNAPK, and cdc2, which are kinase motifs required for DNA repair and cell cycle, were found in the three protein sequences. However, predicted PKG, CKI, p38MAPK, and cdk5 kinase motifs were only found in FmNanos and FmNASP. FmCyclinB also contained the INSR kinase motif similar to FmNanos and EFGR kinase motif in FmNASP. Notably, the predicted RSK and SRC kinase motifs were only found in FmCyclinB and FmNanos, respectively.

**Figure 2:**
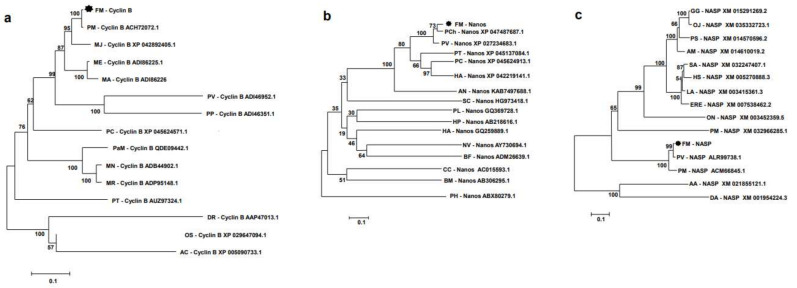
Phylogenetic tree analysis of banana prawn genes with that of other species. (**a**) *FmCyclinB*, (**b**) *FmNanos*, and (**c**) *FmNASP*. Phylogenetic was tree constructed using MEGA with Neighbor-Joining method based on the Poisson model. Values at the node represent the percentage of times that the particular node occurred in 1000 trees generated by bootstrapping the original aligned protein sequences. The asterisk mark (*) is for *F. merguiensis* in this study. Abbreviations: FM (*Fenneropenaeus merguiensis*), PM (*Penaeus monodon*), MJ (*Marsupenaeus japonicus*), ME (*Metapenaeus ensis*), MA (*M. affinis*), PV (*P. vannamei*), PP (*P. penicillatus*), PC (*Procambarus clarkia*), PaM (*Palaemon modestus*), MN (*Macrobrachium nipponense*), MR (*Macrobacium rosenbergii*), PT (*Portunus trituberculatus*), DR (*Danio rerio*), OS (*Octopus sinesis*), AC (*Aplysia californica*), PCh (*P. chiensis*), HA (*Homarus americanus*), AN (*Armadillidium nasatum*), SC (*Sycon ciliatum*), PL (*Pristina leidyi*), HP (*Hemicentrotus pulcherrimus*), HA (*Haliotis asinina*), NV (*Nematostella vectensis*), BF (*Branchiostoma floridae*), CC (*Caligus clemensi*), BM (*Bombyx mori*), PH (*Parhyale hawaiensis*), GG (*Gallus gallus*), OJ (*Oxyura jamaicensis*), PS (*Pelodiscus sinensis*), AM (*Alligator mississippiensis*), SA (*Sapajus apella*), HS (*Homo sapiens*), LA (*Loxodont Africana*), ERE (*Erinaceus europaeus*), ON (*Oreochromis niloticus*), PMa (*Petromyzon marinus*), AA (*Aedes aegypti*), DA (*Drosophila ananassae*).

**Figure 3:**
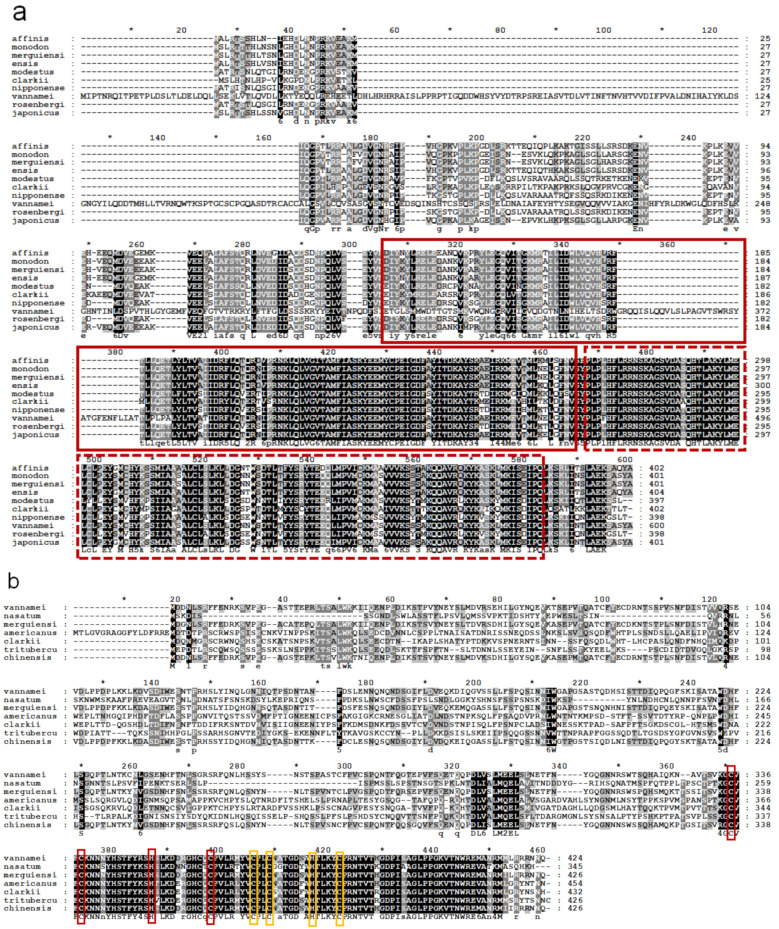

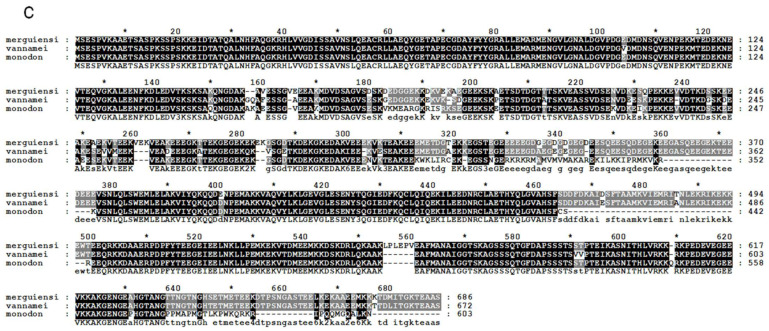
Multiple sequence alignment of deduced protein sequences of *FmCyclinB*, *FmNanos*, and *FmNASP* genes. (**a**) Multiple sequence alignment of banana prawn cyclin B amino acid with that of known crustacean cyclin B amino acid sequences. Red line and dot boxes show two signature cyclin superfamily motifs. The GenBank accession numbers of the cyclin B amino acids are as follows: merguiensi (*Fenneropenaeus merguiensis*), monodon (*Penaeus monodon*, XP_037794731.1), japonicus (*Marsupenaeus japonicus*, XP_042892405.1), ensis (*Metapenaeus ensis*, ADI86225.1), affinis (*Metapenaeus affinis*, ADI86226), clarkii (*Procambarus clarkii*, XP_045624571.1), nipponense (*Macrobachium nipponense*, ADB44902.1), rosenbergi (*Macrobacium rosenbergii*, ADP95148.1), modestus (*Palaemon modestus*, QDE09442.1), and vannamei (*Penaeus vannamei*, ACI46952.1). (**b**) Multiple sequence alignment of Nanos amino acid sequence with known crustacean Nanos amino acid sequences. Red and yellow boxes show two specific conserved Cys-Cys-His-Cys zinc finger motifs of Nanos. The GenBank accession numbers of the Nanos amino acids are as follows: merguiensi (*Fenneropenaeus merguiensis*), chinensis (*Penaeus chinensis*, XP_047487687.1), tritubercu (*Portunus trituberculatus*, XP_045137084.1), nasatum (*Armadillidium nasatum*, KAB7497688.1), and americanus (*Homarus americanus*, XP_042219141.1). (**c**) Multiple sequence alignment analysis of banana prawn *NASP* amino acid sequence with known crustacean *NASP* amino acid sequences. The GenBank accession numbers of the *NASP* amino acid sequences are as follows: merguiensi (*Fenneropenaeus merguiensis*), vannamei (*Penaeus vannamei*, ALR99738.1), and monodon (*Penaeus monodon*, ACM66845.1). NASP, nuclear autoantigenic sperm protein. The amino acid sequences were aligned using BioEdit and represented using GenDoc. Gaps that were introduced to maximize sequence homology are indicated by dashes. Shaded boxes indicate the conserved sequence. The asterisk is counting every 10 amino acid.

The deduced amino acid sequences of banana prawn were aligned with those of other organisms, and phylogenetic trees were constructed, which showed that FmCyclinB (Figure 2a), FmNanos (Figure 2b), and FmNASP (Figure 2c) were clustered in the shrimp group. FmCyclinB was closest to Cyclin B of *P. monodon* (accession no. ACH72072.1), FmNanos was closest to Nanos of *P. chinensis* (accession no. XP_047487687.1), and FmNASP was closest to NASP of *P. vannamei* (accession no. ALR99738.1). Moreover, the amino acid sequence of the banana prawn was analyzed for multiple sequence alignment with other shrimp species. The FmCyclinB sequence alignment showed a highly conserved region with almost all shrimp species except *P. vannamei* (ACI46952.1; Figure 3a). This conserved region is the cyclin superfamily motif. The FmNanos sequence alignment showed a highly conserved region only in two Cys-Cys-His-Cys zinc-finger motifs at a carboxyl side as well (Figure 3b), and these are hallmarks for Nanos proteins [21]. The FmNASP sequence alignment showed a highly conserved region with almost all species (Figure 3c) except for *P. monodon* (accession no. ACM66845.1).

### 3.3. Characterization of Banana Prawn Ovarian Cells

**Figure 4:**
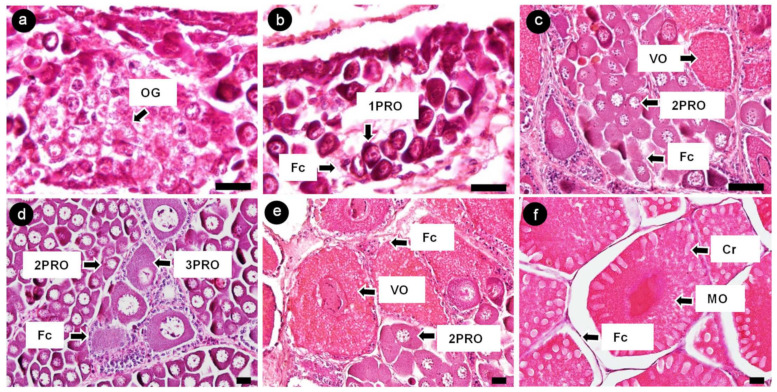
Histological analysis of banana prawn ovaries visualized by hematoxylin and eosin staining. (**a**) OG, (**b**) 1PRO, (**c**) 2PRO, (**d**) 3PRO, (**e**) VO, (**f**) MO, Fc, and Cr (whitehead arrow line). Scale bars represent 20 µM. 1PRO, stage 1 previtellogenic oocyte; 2PRO, stage 2 previtellogenic oocyte; 3PRO, stage 3 previtellogenic oocyte; VO, vitellogenic oocyte; MO, mature oocyte; Fc, follicle cell; Cr, cortical rod.

The ovaries of banana prawns were observed, and oocytes were characterized using a histological method. Oocytes were classified into six stages according to the morphology of the nucleus, oocyte size, and granulosa cell layer [22,23]. Oogonia (OG) were 8–10 µm in diameter and shown as a large nucleus located at the center, surrounded by a thin layer of cytoplasm. The nucleus contained dispersed chromatin (Figure 4a). The first-stage previtellogenic oocytes (1PROs) were 10–20 µm in size and oval shaped; they showed a relatively thicker cytoplasm layer, which has a basophilic property, and more nucleoli appeared at the periphery of nucleus. Moreover, 1PRO was accompanied by follicle cells (Figure 4b). The second-stage previtellogenic oocytes (2PROs) were 20–90 µm in size and had a polygonal cell shape; they contained a peri-nucleolus and were accompanied by follicle cells (Figure 4c). The third-stage previtellogenic oocytes (3PROs) were 80–160 µm is size and had a large polygonal cell shape; the cytoplasm retained the basophilic property. Follicle cells differentiated into a granulosa layer and a theca layer around each cell of the 3PROs (Figure 4d). The vitellogenic oocytes (VOs) were 160–400 µm in size; the cytoplasm showed vitellogenesis with an eosinophilic property and was surrounded by a granulosa layer (Figure 4e). The mature oocytes (MOs) were 400 µm in size at the yolk stage (Figure 4f).

### 3.4. Localization of mRNA-Positive Cells in Ovaries of Banana Prawn via ISH Analysis

**Figure 5:**
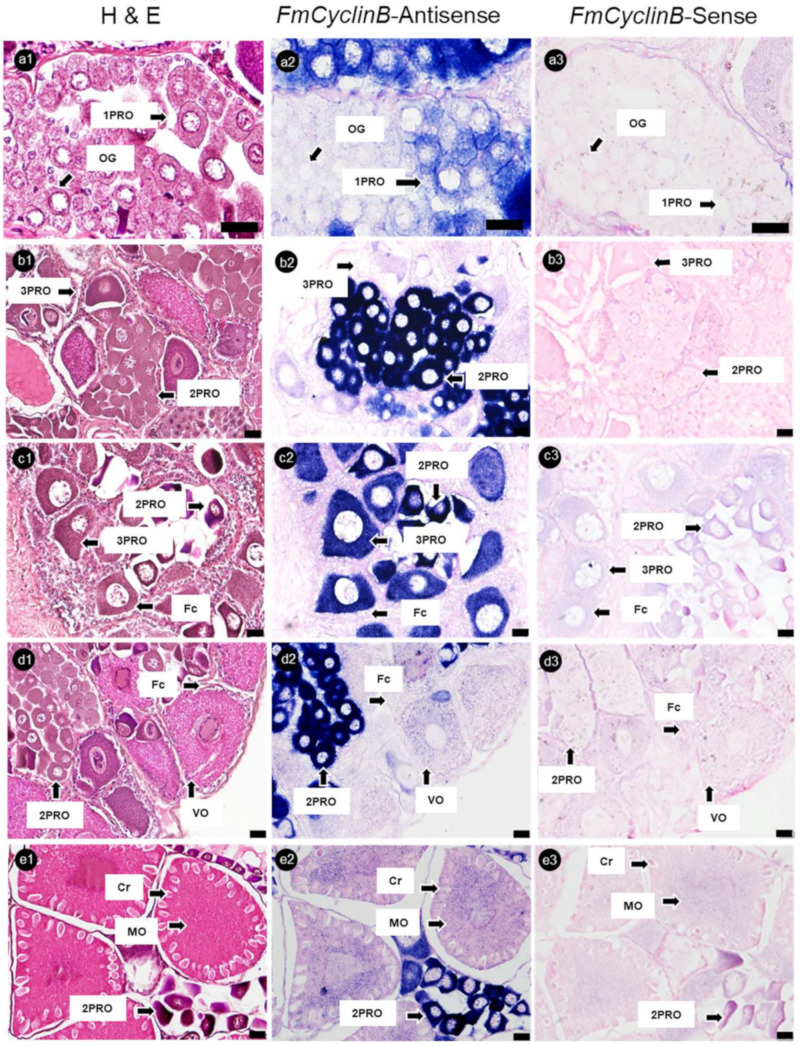
Histological characterization and cyclin B mRNA expression in ovaries of banana prawn, using ISH. Sequential sections of the ovary were divided and stained with (**a1**–**e1**) H&E for representative cell characteristics and hybridized with (**a2**–**e2**) cyclin B antisense RNA probe and (**a3**–**e3**) sense RNA probes. Scale bars represent 20 µM. OG, oogonia; 1PRO, stage 1 previtellogenic oocyte; 2PRO, stage 2 previtellogenic oocyte; 3PRO, stage 3 previtellogenic oocyte; VO, vitellogenic oocyte; MO, mature oocyte; Fc, follicle cell; Cr, cortical rod; H&E, hematoxylin and eosin.

Banana prawn ovary sections were hybridized with *FmCyclinB*, *FmNanos*, and *FmNASP* RNA-probes. The ISH analysis showed that *FmCyclinB* -antisense probes were detected as positive strong signals in the cytoplasm of 1PROs, 2PROs, and 3PROs. Overall, 2PROs showed the most intensive F*mCyclinB*-antisense probe signal, followed by those in 3PROs and 1PROs, while VOs and MOs, which were in the vitellogenic stage, showed a weak signal. Moreover, OG did not show a positive signal of the *FmCyclinB*-antisense probe (Figure 5).

**Figure 6:**
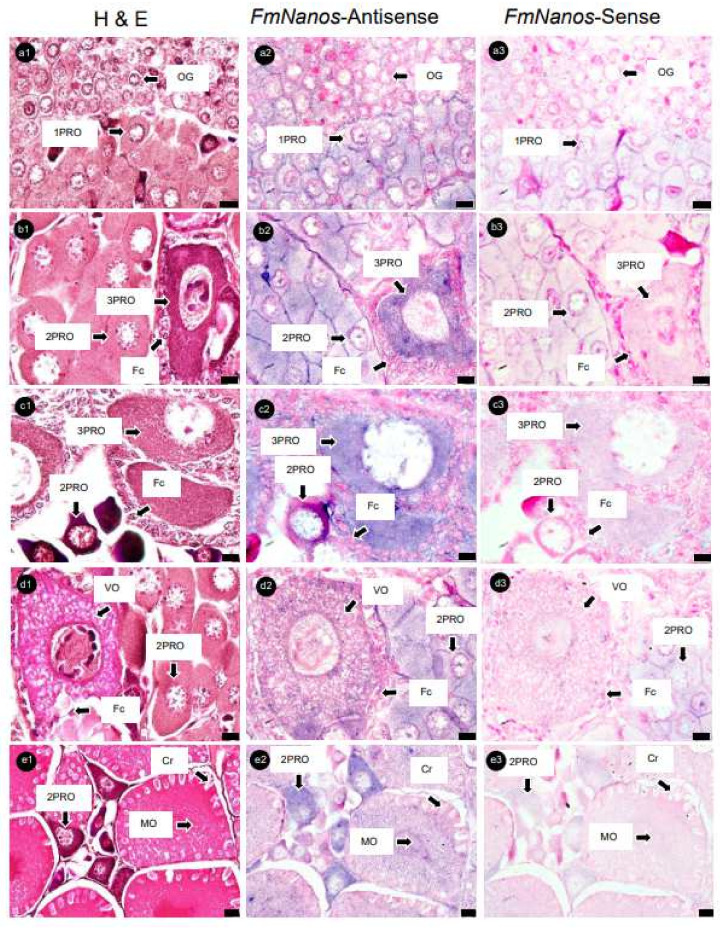
Histological characterization and *Nanos* mRNA expression in ovaries of banana prawn, using ISH. Continuous ovary sections were separated, stained with (**a1**–**e1**) H&E, and then used as a reference cell for comparison with sections hybridized with (**a2**–**e2**) *Nanos* antisense and (**a3**–**e3**) sense RNA probe. Scale bars represent 10 µM in (**a**–**d**) and 20 µM in (**e**). OG, oogonia; 1PRO, stage 1 previtellogenic oocyte; 2PRO, stage 2 previtellogenic oocyte; 3PRO, stage 3 previtellogenic oocyte; VO, vitellogenic oocyte; MO, mature oocyte; Fc, follicle cell; Cr, cortical rod; H&E, hematoxylin and eosin.

**Figure 7:**
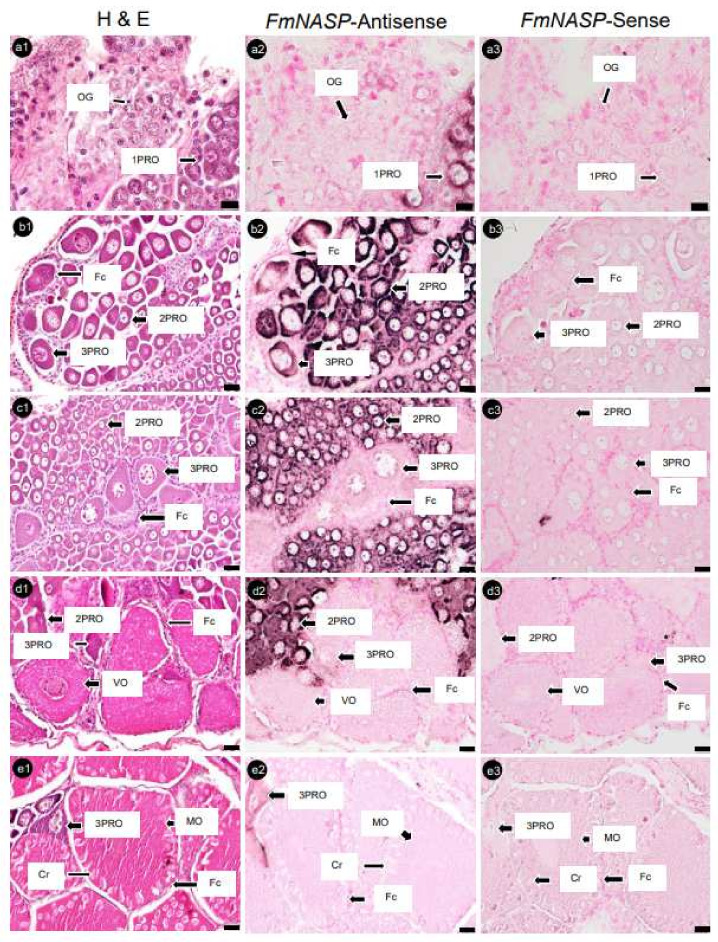
Histological characterization and *NASP* mRNA expression in banana prawn ovaries, using ISH. Serial ovary sections were sorted for staining with (**a1**–**e1**) H&E as reference ovarian cells and hybridized with (**a2**–**e2**) *NASP* antisense and (**a3**–**e3**) sense RNA probes. Scale bars represent 20 µM. *NASP*, nuclear autoantigenic sperm protein; OG, oogonia; 1PRO, stage 1 previtellogenic oocyte; 2PRO, stage 2 previtellogenic oocyte; 3PRO, stage 3 previtellogenic oocyte; VO, vitellogenic oocyte; MO, mature oocyte; Fc, follicle cell; Cr, cortical rod; H&E, hematoxylin and eosin.

Serial ovary sections hybridized with *FmNanos*-antisense probes showed the strongest positive signals in 3PROs, while 1PROs, 2PROs, VOs, and MOs showed slightly positive signals. However, no positive signal was observed in OG (Figure 6). An analysis of *FmNASP*-antisense probes hybridized with ovarian sections revealed positive signals in PROs alone; the most intensive signal to the least intensive signal followed the order 2PRO, 1PRO, and 3PRO. In contrast, a positive signal was not found in OG, VOs, and MOs (Figure 7). The positive signal of each antisense RNA probe was not observed in Fcs and somatic tissues around ovarian germ cells, and sense RNA-probe signals were not observed in any ovary sections.

### 3.5. mRNA Expression Determined via qRT-PCR

**Figure 8:**
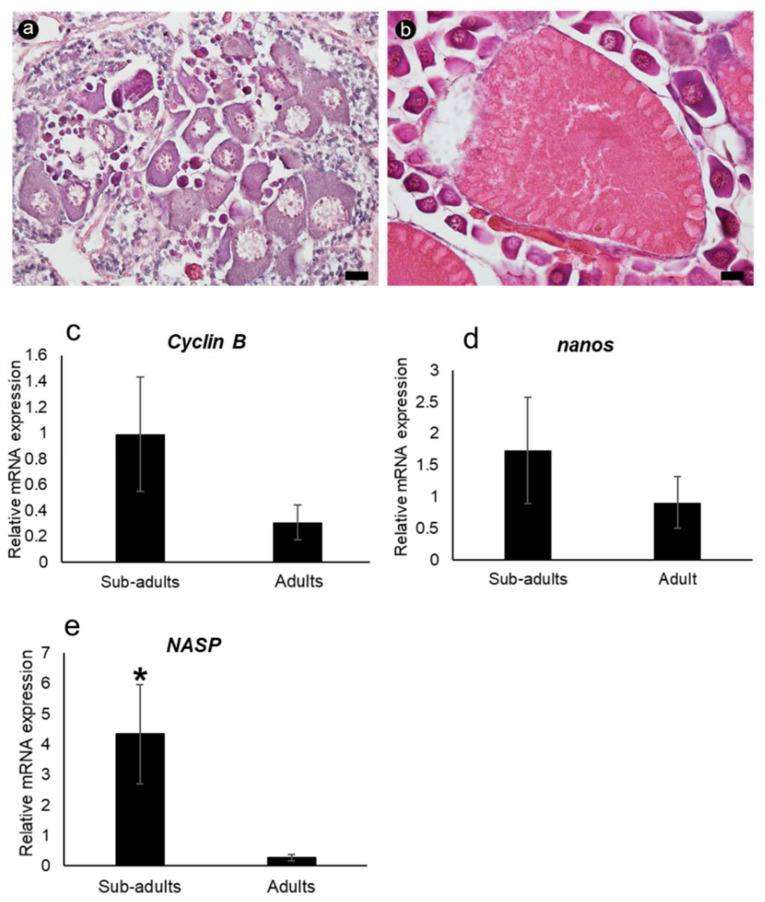
Relative expression levels of ovarian germ cell-specific genes in sub-adult and adult ovaries, using qRT-PCR. Sections stained with hematoxylin and eosin were used for characterizing (**a**) sub-adult and (**b**) adult ovaries. Scale bar: 20 µM. Relative mRNA expression levels of (**c**) *Cyclin B*, (**d**) *Nanos*, and (**e**) *NASP*, respectively, between sub-adult and adult ovaries. The mRNA expression level of each gene was normalized to the expression level of the internal standard beta-actin. Asterisk indicates significant differences in relative mRNA expression level (*p* ≤ 0.05). *NASP*, nuclear autoantigenic sperm protein.

The relative mRNA expression level of each gene was determined via qRT-PCR in the ovarian tissues of sub-adult and adult banana prawns (Figure 8). The results were normalized to the expression level of the internal standard beta-actin (mean ± SD, *N* = 5 and *p* < 0.05). The mRNA expression levels of *FmCyclinB* and *FmNanos* were slightly higher in sub-adult prawns than in adult prawns. *FmNASP* expression was significantly higher in sub-adult shrimps. This result supports the ISH results that *FmNASP* expression was only detected in pre-vitellogenic stages.

## 4. Discussion

In this study, we identified germ cell-specific genes from the ovarian transcriptome of banana prawns. Three genes were expressed only in the gonads, namely *FmCyclinB*, *FmNanos*, and *FmNASP*. We characterized *FmCyclinB*, *FmNanos*, and *FmNASP* via sequence and mRNA expression analyses. ISH analyses revealed that the three genes showed different expression patterns in the ovaries. *FmCyclinB* and *FmNanos* were expressed throughout oogenesis, whereas *FmNASP* was highly expressed during pre-vitellogenesis alone.

*FmCyclinB* full length has a nucleotide sequence of 2420 bp, encoding a polypeptide of 401 amino acids. Two conserved signature motifs of cyclin B super family were identified in FmCyclinB-deduced amino acid sequence. The cyclin super family motifs are cyclin the destruction box, which signals for degradation of cyclin B at the transition from metaphase to anaphase [24]. The cyclin B gene in most eukaryotic cells is expressed during the G2/M phase [25]. The expression of cyclin B is highly related to the cell proliferation status [26]. In this study, *FmCyclinB* was highly expressed in pre-vitellogenic oocytes (PROs) during oogenesis. *FmCyclinB* was initially expressed in early pre-vitellogenic oocytes (1PROs), and the expression level gradually decreased in late pre-vitellogenic oocytes (3PROs). By the middle pre-vitellogenic oocytes (2PROs) stage, the strongest accumulated signal was observed, and *FmCyclinB* still showed a faint signal in vitellogenic oocytes (VOs). The PROs that accumulated with expressed FmCyclin B are the cells which have proliferation status. Our findings of *FmCyclinB* expressed in PROs were similar to cyclin B1 gene expression detected in *M. japonicus* via ISH [27]. In *M. japonicus*, *cyclin B1* gene expression is observed in PROs, and it accumulates at the late stage of PROs. However, no expression of *cyclin B1* is detected in Vos, thus contradicting our finding. The *FmCyclinB* expression at the late stages of oocyte development may play a role similar to that of *cyclin B* in *Drosophila* [28] and *Xenopus* [29] that has been reported as a maternal mRNA. The maternal mRNA is generally transcribed at early stage of oogenesis in oviparous animals and required in germline development [30]. The 3′ UTR of maternal mRNAs contains cytoplasmic polyadenylation elements [31], which are associated with dramatic changes of translational during meiotic maturation and/or early embryogenesis [32]. Moreover, alteration in the primary structure of the maternal mRNA transcript may be related to translation control [33]. In this study, *FmCyclinB* was identified three polyadenylation motifs (AATAAA) in 3′ UTR, meaning that the *FmCyclinB* transcript had three forms. This finding is similar to that of *M. japonicus* [27] but different from that of *P. monodon* [34], which has only one form. However, the abundant expression of *FmCyclinB* transcripts via qRT-PCR in adult ovaries is greater than that in sub-adult ovaries, similar to *P. monodon* [10]. In *P. monodon*, *cyclin B* in broodstock ovaries was reported to be involved in oogenesis and/or ovarian development more than in sub-adults. Moreover, our subtype characterization of pre-vitellogenesis revealed relatively greater dynamic expression of *FmCyclinB*. 

*FmCyclinB* expression was detected throughout oogenesis, similar to *FmNanos*. The *FmNanos* full length has a nucleotide sequence of 1486 bp and deduced a peptide of 426 amino acids. For the FmNanos-deduced amino acid sequence, researchers observed two conserved zinc-finger motifs (Cys-Cys-His-Cys) at the C-terminal region, which is necessary for nanos function [35]. *FmNanos* was expressed at almost the same level throughout oogenesis. The results were similar to Nanos protein expression in all stages of *Bombyx mori* oocytes, which was analyzed via immunohistochemistry [36]. Previously, Nanos RNA has been detected in the early stages of embryos in *Drosophila* [37], *Apis mellifera* [38], and *B. mori* [36] and plays a role as maternal effector molecules, which are required for germ cell gene expression and localization in embryogenesis. Based on these reports, we speculated that *FmNanos* expression accumulated in mature oocytes (MOs) may play a role as maternal molecules as well. 

*FmCyclinB* and *FmNanos* both are maternal molecules which are expressed during oogenesis. The highest expression level of *FmCyclinB* was detected in 2PROs, while that of *FmNanos* was detected in 3PROs. From these results, we suggested that the increase in *FmNanos* expression level in 3PROs may suppress *FmCyclinB* expression before VO development, which was supported by previous findings in *Drosophila* [38] and *Xenopus* [29]. Nanos protein functions, together with Pumilio protein as a translation regulator, were required to suppress maternal *cyclin B* mRNA expression and delay cell cycle during oocyte maturation [39]. 

*FmNASP*, at full length, is 2700 nucleotides long and is a polypeptide with 686 amino acids. The *FmNASP* expression was found only in PROs with the highest signal in 1PRO and 2PRO and rapidly decreased in 3PROs. These results were supported by the qRT-PCR results that showed a significantly higher expression of *FmNASP* in sub-adult ovaries than in adult ovaries, which contained most of the PROs. These findings suggest that *FmNASP* may be specifically involved in the proliferation process of oocytes during oogenesis. However, these results contradict those of [28], who reported that the NASP expression level in *P. monodon* is significantly increased in Mos, and the expression level in adults is greater than that in sub-adults, based on microarray and qRT-PCR. The results from qRT-PCR did not clearly explain which stage of the ovarian germ cell contained NASP expression because each stage of the shrimp ovary contains various types of ovarian germ cells. Especially, genes expressed in PROs could be found in every stage of ovarian development. Thus, our results from ISH explained the more precise trends in expression. Moreover, the multiple sequence alignment of NASP amino acid sequences showed that amino acids at 320–380 of FmNASP differed from those in the *P. monodon* sequence. Moreover, amino acids at 460–500 and 640–680 showed a gap in the NASP sequence of *P. monodon*. Both the different sequence and gap predicted the serine and tyrosine phosphorylation residues and ATM, PKC, CKI, CKII, cdc2, and DNAPK motifs. From these differences, we suggested that the *NASP* genes of banana prawn and *P. monodon* may play different roles during oogenesis. 

## 5. Conclusions

We found three gonad-specific gene markers. Based on ISH, we evaluated the expression and localization of these genes in banana prawns’ ovarian germ cells. Our finding will assist studies on the molecular mechanisms of ovarian-cell gene regulation during oogenesis and female reproduction in the banana prawn.

## Figures and Tables

**Table 1:** Primers used for amplifying genes in organs.

Gene Name	Primer Name	Primer Sequence	Annealing Temp. and Time	Elongation Temp. and Time
Cyclin A	*FmCyclinA*-F	5′-GCTGCGAAATATGAGGAGAT-3′	56 °C, 45 s	72 °C, 45 s
	*FmCyclinA*-R	5′-TATGGCAGTTCAAACGCTA-3′		
Cyclin B	*FmCyclinB*-F	5′-GATGTGGAGGAGGAAGC-3′	60 °C, 30 s	72 °C, 30 s
	*FmCyclinB*-R	5′-AGGAAGTGCAAGGGAAGG-3′		
Cyclin E	*FmCyclinE*-F	5′-CTGTGGCTATGCTCACTCCA-3′	60 °C, 30 s	72 °C, 30 s
	*FmCyclinE*-R	5′-GGAGGAACCTGAATGACAAT-3′		
Nanos	*FmNanos*-F	5′-ATTGCCAGAAATCACCAG-3′	59 °C, 30 s	72 °C, 30 s
	*FmNanos*-R	5′-ATTCTGCCGTGTCAACAT-3′		
*NASP*	*FmNASP*-F	5′-AAGGTTGAGGCTAAGGAG-3′	62 °C, 45 s	72 °C, 45 s
	*FmNASP*-R	5′-CACAGGTTCAAGAGGCAG-3′		
*MARF1*	*FmMARF1*-F	5′-TGAGGCTTTTCCAGTTGCTT-3′	64 °C, 45 s	72 °C, 45 s
	*FmMARF1*-R	5′-TAGGGGATAGGTGCAGTTGG-3′		
Actin	*Actin*-F	5′-GCTACAGCTTCACCACCACCG-3′	58 °C, 30 s	72 °C, 30 s
	*Actin*-R	5′-GATGTCCACGTCRCACTTCAT-3′		

**Table 2:** Primers for real-time polymerase chain reaction.

Gene Name	Primer Name	Primer Sequence	Annealing Temp. and Time
Cyclin B	q*FmCyclinB*-F	5′-GGAAGTGGTAGAGCATGTGGAGCA-3′	67 °C, 30 s
	q*FmCyclinB*-R	5′-TTGAAGCAGGGTGAAGCGGAGG-3′	
Nanos	q*FmNanos*-F	5′-GGCAGTCCTCAGGATACATTTCAGC-3′	67 °C, 45 s
	q*FmNanos*-R	5′-GCACAGAGGGCAGACATACATTC-3′	
*NASP*	q*FmNASP*-F	5′-TGCCGAGACCAGTGCCAGCC-3′	67 °C, 30 s
	q*FmNASP*-R	5′-CTCCGTTCTCCATGCGTGCCA-3′	
Actin	*Actin*-F	5′-GCTACAGCTTCACCACCACCG-3′	54 °C, 30 s
	*Actin*-R	5′-GATGTCCACGTCRCACTTCAT-3′	

**Table 3:** Prediction of sequence properties.

Gene	Nucleotide (bp)	Amino Acid (Residue)	Phosphorylation Residue	Kinase Motif
*FmCyclinB*	2420	401	S = 23, T = 7, Y = 7	PKA, PKC, CKII, ATM, DNAPK, RSK, INSR, EGFR, cdc2
*FmNanos*	1486	426	S = 36, T = 14, Y = 3	PKA, PKC, PKG, CKI, CKII, ATM, DNAPK, SRC, INSR, p38MAPK, cdc2, cdk5
*FmNASP*	2700	686	S = 44, T = 32, Y = 6	PKA, PKC, PKG, CKI, CKII, ATM, DNAPK, EGFR, p38MAPK, cdc2, cdk5

*FmNASP*, banana-prawn-derived nuclear autoantigenic sperm protein.

## Data Availability

The full-length cDNA sequences of *CyclinB*, *Nanos*, and *NASP* of *F. merguiensis* were deposited at GenBank, under the accession numbers OP156936, OP296393, and OP156937, respectively.

## Supplementary Materials

The following supporting information can be downloaded at https://www.mdpi.com/article/10.3390/cimb45070360/s1, Figure S1: title; The full-length cDNA from in silico analysis, deduced protein sequences of *FmCyclinB*, *FmNanos*, and *FmNASP* genes.
