# Supplementary material for: Molecular Markers of Ovarian Germ Cells of Banana Prawn (Fenneropenaeus merguiensis)
Source: Curr Issues Mol Biol. 2023 Jul 7;45(7):5708–24. doi: 10.3390/cimb45070360 (PMC10378296; doi:10.3390/cimb45070360)
Supplement: Supplementary file 1 [file cimb-45-00360-s001.zip › cimb-2471662-supplementary.pdf]

a

```
1     tcg agt cgg cgt cgc ttg aga gag gac ctg ttt tga gga gat ttc   45
46    aca gtg aaa aaa gcc aca cat tct tga ata tcc ggt tga ttt ttg   90
91    tcc atc atg tct ttg aga act acc aca cat ctc acc agt aac ctg   135
1              M   S   L   R   T   T   T   H   L   T   S   N   L   13

136   ggg cac gac ctg aac aac ccc cgc aaa gta gag gcc aag atg atc   180
14     G   H   D   L   N   N   P   R   K   V   E   A   K   M   I    28

181   cag ggg cca gtc acc cgt cgt gct ttc gtg gat gtt ggc aac cgt   225
29     Q   G   P   V   T   R   R   A   F   V   D   V   G   N   R    43

226   gcc att cct gtg caa ggg ccc aag gct ccc ctc aag ccc ggg gag   270
44     A   I   P   V   Q   G   P   K   A   P   L   K   P   G   E    58

271   atc tcc cgt aat gag tcc gtg aaa ctg cag aag ccc aaa gcc ggc   315
59     I   S   R   N   E   S   V   K   L   Q   K   P   K   A   G    73

316   ctc tct ggg ctg cac gcc aga tcc ggc aaa gag aat gtg aag ccc   360
74     L   S   G   L   H   A   R   S   G   K   E   N   V   K   P    88

361   ctg aag gaa gtg gta gag cat gtg gag cag atg gat gtg gag gag   405
89     L   K   E   V   V   E   H   V   E   Q   M   D   V   E   E    103

406   gaa gcc aag gtg gaa gaa ctg gcc att gct ttc tcc acc cag aga   450
104    E   A   K   V   E   E   L   A   I   A   F   S   T   Q   R    118

451   cta gat gtt gaa gat att gat tcc caa gac agt gat aat cct cag   495
119    L   D   V   E   D   I   D   S   Q   D   S   D   N   P   Q    133

496   ctt gta tct gaa tat gtg aat gat atc tac aag tac ttg cga gag   540
134    L   V   S   E   Y   V   N   D   I   Y   K   Y   L   R   E    148

541   ctg gag gat gcc aac aaa gtc aag gcc aga tac cta gaa ggc caa   585
149    L   E   D   A   N   K   V   K   A   R   Y   L   E   G   Q    163

586   gta att aca gga aag atg agg gca att ttg att gat tgg ctt gtc   630
164    V   I   T   G   K   M   R   A   I   L   I   D   W   L   V    178

631   caa gtc cac ctc cgc ttc acc ctg ctt caa gag aca ctg tat ctg   675
179    Q   V   H   L   R   F   T   L   L   Q   E   T   L   Y   L    193

676   act gtt gct atc att gac aga ttt ctc cag act cag agg aat ata   720
194    T   V   A   I   I   D   R   F   L   Q   T   Q   R   N   I    208

721   cca cgt aac aag cta cag tta gtt ggt gtg act gcc atg ttt att   765
209    P   R   N   K   L   Q   L   V   G   V   T   A   M   F   I    223

766   gct agc aaa tat gaa gaa atg tat tgc cca gaa atc ggg gac ttc   810
224    A   S   K   Y   E   E   M   Y   C   P   E   I   G   D   F    238

811   gca tac atc aca gac aaa gcc tac tca aag gca gag att cgt aaa   855
239    A   Y   I   T   D   K   A   Y   S   K   A   E   I   R   K    253
```

856 atg gag gtg acc atg ctg aat gag ctg ggc ttc aat gta tcc tat 900
254 M E V T M L N E L G F N V S Y 268
901 ccc ctt ccc ttg cac ttc ctg cga aga aac agc aaa gct ggc tct 945
269 P L P L H F L R R N S K A G S 283

946 gtt gat gct tct caa cat acc ttg gca aag tac ctg atg gaa ctt 990
284 V D A S Q H T L A K Y L M E L 298

991 tgc ttg ccc gag tac agc atg tgc cat tac aaa tcg tca atg att 1035
299 C L P E Y S M C H Y K S S M I 313

1036 gct gca tct gct ctc tgc ctt tca ctt aag ttg ctg gat ggc aat 1080
314 A A S A L C L S L K L L D G N 328

1081 aac tgg agc gat aca ttg act ttc tat tct cgc tac act gaa caa 1125
329 N W S D T L T F Y S R Y T E Q 343

1126 cag ctt atg cca gtc atg tgc aaa atg gca gca gtt gta gtt aag 1170
344 Q L M P V M C K M A A V V V K 358

1171 agc agt agt gcc aag caa cag gct gta aga cag aag tac aaa gcc 1215
359 S S S A K Q Q A V R Q K Y K A 373

1216 agc aag ttg atg aag att agt gag att ccc cag ctc aag tca aag 1260
374 S K L M K I S E I P E L K S K 388

1261 ctc atc aac tcg ctc gca gag aag agt gcg tct tat gca tga ggt 1305
389 L I N S L A E K S A S Y A - 401

1306 ggc tgc cat tta taa agt aaa tat tgt aca tgt tga atg aat gga 1350
1351 ctg gtt ttt gta cat att act tta aga tgc cag ttt gtt ttc ttt 1395
1396 ttc ata agc ttt cag att tat aaa ata ctg act acc agt att taa 1440
1441 ctt ttt att tta caa gac tag ttc tct ggg tta gca tgt aca acc 1485
1486 cag agt gac tgg ttt tgg cat cta tcc att tat cat ggt ctt cat 1530
1531 tgt att aat ata att tta gac agg aca aaa atg gct att tga taa 1575
1576 aag gaa ctt gca aag aag tat atg cta taa tgt ctt gta atg tat 1620
1621 ata tga tct gtg ata aac ttt tag tta cgt tct gta tgt ata cct 1665
1666 ata taa taa acc agt ttt atc caa gat ctg tat tcc act att ttt 1710
1711 tat gcc ttt agt tat aca ttg caa cca tat ttg tgg att gac agt 1755
1756 aaa taa gat gat tta tgg att aat gtt cat aac cat tcc ttt cct 1800
1801 ata gtt ttc agc atc acc aaa gaa aat aaa tat gca tgc ttt act 1845
1846 tta taa atg caa aag aac agg ctt gtg tgc caa ata ttg tac aca 1890
1891 ttt tat tct tta ttt tag aac cca gat gtt gat ttt atg tgc agg 1935
1936 atg cat att cct tta tgc aga tta ggt gct ttg cca aat gta att 1980
1981 tta act tct gca ctt tta aat att ccg taa att gtc aca ggt ata 2025
2026 ttt ttt gta tgg taa tgc agt ttt cgt aat tta gga tta atg aac 2070
2071 caa gga atc ctg ttt acc att att ttt gtt gta cct ttt tga tca 2115
2116 gct gct tta ggt ggt agt ggt ccc aaa act aca agg gac aaa gat 2160
2161 gga cgt tgt att tag cgg ctg taa aat agg ata gac ttg gag aaa 2205
2206 aga gga aaa atg gaa cta ttt gtt cta gtg ttg cat cct aga aga 2250
2251 gat cta gtt ctt gac caa agg atg tat taa att gtt ctc atg tgg 2295
2296 att tga cta gac taa acc tag gtg cta tat tct tac tat gta tac 2340
2341 ttc cag ttg aaa gaa atg taa atg cat gtt acc cat act gta ata 2385
2386 ttt ttc aga aaa gta aac gaa ata caa taa aaa 2418

b

```
2    tca gtt gaa cga cga aag gag aaa ctt ttt aaa act caa tta taa   46
47   ctt ctc att gcg aaa atg gac ggc aac tta tca cgt ttt ttt gaa   91
1                         M   D   G   N   L   S   R   F   F   E    10

92   gat agg aaa tct gtg cca gaa ggt gct tgt gct aca gaa ccc cag   136
11    D   R   K   S   V   P   E   G   A   C   A   T   E   P   Q    25

137  ctg aca agt gca cta tgg aaa aaa att att cag gaa aat cct tta   181
26    L   T   S   A   L   W   K   K   I   I   Q   E   N   P   L    40

182  gat att aaa agt act tct gtg tat aat gaa tat agt tta acg gat   226
41    D   I   K   S   T   S   V   Y   N   E   Y   S   L   T   D    55

227  gtt aga agt gac cat att cta gga tat agc caa gag gtt aaa gca   271
56    V   R   S   D   H   I   L   G   Y   S   Q   E   V   K   A    70

272  agt gaa cca gtg act caa gca aca tgt ttt tat gaa tgc gat aaa   316
71    S   E   P   V   T   Q   A   T   C   F   Y   E   C   D   K    85

317  aat aca agt acc cca tta tca aac ttt gat ata agt aca gtg tta   361
86    N   T   S   T   P   L   S   N   F   D   I   S   T   V   L    100

362  cac aga aat gag gtg gat tta cct cct gat cct ttt aag aaa ttg   406
101   H   R   N   E   V   D   L   P   P   D   P   F   K   K   L    115

407  aaa gat gca agt gat atc tgg gaa tca agt aca cca aga cat tca   451
116   K   D   A   S   D   I   W   E   S   S   T   P   R   H   S    130

452  tta tat att gac caa cat ggt aac aat agt caa aca gca agt gat   496
131   L   Y   I   D   Q   H   G   N   N   S   Q   T   A   S   D    145

497  aat aca att acc ttt gat agt ttc gaa agt aac cag agt cag aat   541
146   N   T   I   T   F   D   S   F   E   S   N   Q   S   Q   N    160

542  gac tct ggt ata tat tta gat gta gag cag aag gag atg caa ggt   586
161   D   S   G   I   Y   L   D   V   E   Q   K   E   M   Q   G    175

587  gcg agt agc ctg tta ttt tca acg cag tct ata aac aat att tgg   631
176   A   S   S   L   L   F   S   T   Q   S   I   N   N   I   W    190

632  ggt gca cct gga agt acc agt aac caa aac cat aat ata tca aca   676
191   G   A   P   G   S   T   S   N   Q   N   H   N   I   S   T    205

677  aca gat att cag cca cag gaa tat agt aag atc agt gca aca gca   721
206   T   D   I   Q   P   Q   E   Y   S   K   I   S   A   T   A    220

722  tgg gat cac ttc ctt agt ggt cag cca aca tta aac acc aaa tat   766
221   W   D   H   F   L   S   G   Q   P   T   L   N   T   K   Y    235

767  aat gta gga agt gac aat cac ttc tct aat ttg tca agc aga agt   811
236   N   V   G   S   D   N   H   F   S   N   L   S   S   R   S    250
```

812 aga ttc cag aac ctc cag tcc aat tac aat aac ttg aca tca cca 856
251 R F Q N L Q S N Y N N L T S P 265
857 gtc aac acc tgt ctt cca gta ggc agt cct cag gat aca ttt cag 901
266 V N T C L P V G S P Q D T F Q 280

902 cga tct gaa cca gta ttc tca caa gaa aat cag caa cca gat tta 946
281 R S E P V F S Q E N Q Q P D L 295

947 gtt tca ctt atg gag gaa ctg tct tta aat gaa aca ttt aat tat 991
296 V S L M E E L S L N E T F N Y 310

992 gga caa gga aac aac aga tca tgg tct ccc caa cat tca atg caa 1036
311 G Q G N N R S W S P Q H S M Q 325

1037 aaa act act agt tcc ata acc tca gtc aag ggt tgt gtg ttt tgt 1081
326 K T T S S I T S V K G C V F C 340

1082 aaa aac aac aat tac cat tct acc ttt tat agg tca cat tca tta 1126
341 K N N N Y H S T F Y R S H S L 355

1127 aag gat gat cgc ggt cat tgc cag tgt cct gta cta aga atg tat 1171
356 K D D R G H C Q C P V L R M Y 370

1172 gtc tgc cct ctg tgc aat gct aca ggg gat tca gca cat acc ctc 1216
371 V C P L C N A T G D S A H T L 385

1217 aaa tat tgc cca agg aat act gta acc cat gga gat cca ata tca 1261
486 K Y C P R N T V T H G D P I S 400

1262 gca ggt ctt ccc cca ggc aaa gtt acc aat tgg aga gag atg gca 1306
401 A G L P P G K V T N W R E M A 415

1307 aat cga atg ata tta cga cga agg aat aat caa taa ata ggc tgt 1351
416 N R M I L R R R N N Q - 426

1352 caa tac cta tga tgt tta tga cat tta tta ttt tga tgt aaa ttt 1396
1397 tat atg gat ctt tga tat ttg atg aaa atc atg gaa aat aat ttt 1441
1442 ctt aga cat ttt gaa cta ttt gtg ttt ctt act ttt atg act 1483

c

2 aaa gaa gcg tca atg gcg tcc agt gtc tcg gcg gga cca cac tct 46
47 ttt ccg gcc ttt gta cgc tcc aca cgc gca acc ccg tca ccc tcg 91
92 ttt gtt gct tcc gaa cgt tac taa cag tcg acc acg atg tcc gag 136
1 M S E 3

137 tcg ccg gta aaa gct gcc gag acc agt gcc agc ccc aag agc tcg 181
4 S P V K A A E T S A S P K S S 18

182 ccg tcc aag aaa gag att gat aca gca acc caa gct tta aat cac 226
19 P S K K E I D T A T Q A L N H 33

227 ttt gct cag ggc aag aga cac ttg gtt gtt ggt gac att tcg tct 271
34 F A Q G K R H L V V G D I S S 48

272 gca gtt aat tct ttg cag gag gca tgt aga cta ctc gca gag caa 316
49 A V N S L Q E A C R L L A E Q 63

317 tac ggt gaa act gct cca gag tgt ggt gat gct tat ttc tac tat 361
64 Y G E T A P E C G D A Y F Y Y 78

362 ggc cgt gca ttg ctt gaa atg gca cgc atg gag aac gga gtc tta 406
79 G R A L L E M A R M E N G V L 93

407 gga aat gct ttg gat gga gtt ccc gat gga gag gac atg gac aat 451
94 G N A L D G V P D G E D M D N 108

452 tcc cag gta gaa aat cct gaa aaa atg aca gag gat gag aag aac 496
109 S Q V E N P E K M T E D E K N 123

497 gag gta aca gaa cag gtt ggg aaa gcg ttg gaa gaa aac ttt aaa 541
124 E V T E Q V G K A L E E N F K 138

542 gat ctt gag gat gtg aca aaa agt aaa tcg gca aag cag aat gga 586
139 D L E D V T K S K S A K Q N G 153

587 gat gca aag gca gtg gag tct tca ggt gtt gag gag gag gct aaa 631
154 D A K A V E S S G V E E E A K 168

632 atg gat gta gat tca gct gga gtg tca gat tcc aaa gat gaa gat 676
169 M D V D S A G V S D S K D E D 183

677 gga ggg gag aag aaa gat aaa gta gag aag gca gaa ggg gag gaa 721
184 G G E K K D K V E K A E G E E 198

722 aag agt aaa gca gaa acc tcg gac act gat ggc acc acc act tcc 766
199 K S K A E T S D T D G T T T S 213

767 aaa gta gag gct agc tca gta gat agt gag aat gta gac aag gaa 811
214 K V E A S S V D S E N V D K E 228

812 agc cag cct gag aaa aag gaa gtt gtg gat acc aaa gat agt tcc 856
229 S Q P E K K E V V D T K D S S 243

857 aaa gag gag gca aag gaa gcg gag aag gtg acg gag gag aag gtt 901
244 K E E A K E A E K V T E E K V 258

902 gag aag gtt gag gct aag gag gaa gaa ggg aaa acc act gag aag 946
259 E K V E A K E E E G K T T E K 273

947 gga gag gga gag aag gaa aag gaa aag gga tca gga gac act aaa 991
274 G E G E K E K E K G S G D T K 288

992 gat gaa aag gga aag gaa gat gcc aaa gtt gaa gag gag aaa gta 1036
289 D E K G K E D A K V E E E K V 303

1037 aaa acg gaa gca aag gaa gag gaa atg gaa act gat ggc act gag 1081
304 K T E A K E E E M E T D G T E 318

1082 aag aag gaa ggc agt aca gaa ggc gag gaa gag gag gaa ggt gat 1126
319 K K E G S T E G E E E E E G D 333

1127 ggt gaa ggc gat ggc gat ggt gaa ggt gat gaa gaa tct caa gaa 1171
334 G E G D G D G E G D E E S Q E 348

1172 gaa tcc cag gat gaa gga gaa aag gag gaa ggt gcc agc cag gaa 1216
349 E S Q D E G E K E E G A S Q E 363

1217 gaa ggg gaa aag act gaa gag gat gag gaa gag gta tca aac ctg 1261
364 E G E K T E E D E E E V S N L 378

1262 cag ctc tct tgg gaa atg ttg gag ttg gca aag gtt atc tat cag 1306
379 Q L S W E M L E L A K V I Y Q 393

1307 aag caa cag gat gag aac cca gag atg gcc aag aaa gtt gcc caa 1351
394 K Q Q D E N P E M A K K V A Q 408

1352 gtc tac cta aaa ctt gga gaa gta ggc ttg gag agt gaa aac tat 1396
409 V Y L K L G E V G L E S E N Y 423

1397 aca cag ggt att gag gac ttc aaa caa tgt ctg caa ata cag gaa 1441
424 T Q G I E D F K Q C L Q I Q E 438

1442 aaa att ctt gag gaa gac aac agg tgt ttg gca gaa acc cat tac 1486
439 K I L E E D N R C L A E T H Y 453

1487 cag ctt ggt gta gca cac tcc ttc tca gat gac ttt gac aag gca 1531
454 Q L G V A H S F S D D F D K A 468

1532 att gat agc ttt aca gca gca atg aag gtt att gaa atg aga att 1576
469 I D S F T A A M K V I E M R I 483

1577 act aat ctt gaa aaa cgg ata aag gag aaa aag gaa tgg aca gaa 1621
484 T N L E K R I K E K K E W T E 498

1622 gag caa aga aag aaa gat gct gca gag aga cct gat cca ttc tat 1666
499 E Q R K K D A A E R P D P F Y 513

1667 aca gaa gaa ggc gag att gaa gaa tta aac aag ttg tta cca gag 1711
514 T E E G E I E E L N K L L P E 528

1712 atg aag gaa aag gtt aca gat atg gag gaa atg aag aaa gac agc 1756
529 M K E K V T D M E E M K K D S 543

1757 aag gac aga ctc cag aaa gca gca aag ctg cct ctt gaa cct gtg 1801
544 K D R L Q K A A K L P L E P V 558

1802 gaa gca ttc atg gca aat gca att ggt ggc acc tcc aaa gct gga 1846
559 E A F M A N A I G G T S K A G 573

1847 tct tca tca caa act gga ttt gat gca cct tca agt tct aca tcc 1891
574 S S S Q T G F D A P S S S T S 588

1892 tca acc ccc aca gaa ata aag gct tcc aac att act cat ctt gta 1936
589 S T P T E I K A S N I T H L V 603

1937 aga aag aag agg aaa cca gaa gat gag gtt gag gga gaa gag gtg 1981
604 R K K R K P E D E V E G E E V 618

1982 aaa aag gca aaa ggc gag aat gga gaa gct cat gga act gct aat 2026
619 K K A K G E N G E A H G T A N 633

2027 gga act acc aat ggc acc aat ggg cac tct gaa acc atg gaa acg 2071
634 G T T N G T N G H S E T M E T 648

2072 gag gaa aag gat acc cct tca aat ggg gca agc act gaa gaa tta 2116
649 E E K D T P S N G A S T E E L 663

2117 aag gag aaa gca gct gaa gag atg aag aaa aag acg gat atg atc 2161
664 K E K A A E E M K K K T D M I 678

2162 act ggg aaa acc gag gca gca tcc tag ata cgt gta taa tgc ctt 2206
679 T G K T E A A S - 686

2207 taa aac act gcc agt tat tgg act aag aac ttt tgt att tta ttg 2251
2252 taa tgg aag cca ttt tat tac cag tga act gtg ttt taa gat tta 2296
2297 cat aat tta tta aaa atg aaa tgt aca ttt ttt ctg ttt cta ccc 2341
2342 aac tac att gaa cat tta ggt atg aag tga ttt ctt gcc ttg gga 2386
2387 ctg att ttt gaa gtg ccc aga tgt ggt gct gct ggt gtg gat ggt 2431
2432 acc tga aat atg aac ttt agt tcg act agt ttc tca att tta aac 2476
2477 ttt ttt tgg cca caa gtt ttg tat tgt aaa ctt tca ata ttt gca 2521
2522 gtt tac tac ctg gaa atc taa cta acc att cct gat ttt aaa tgt 2566
2567 ttt agt tga gat tta tat caa atg ctg aat gtt tat ttc tgc cta 2611
2612 aaa cat gga att taa agg aaa atg aaa tgc aat tac aac tgc ttt 2656
2657 att tga gga ata taa ttc taa aca tgt aat cac aat gca gca 2698

Figure S1. The full-length cDNA from *in silico* analysis, deduced protein sequences of *FmCyclinB*, *FmNanos*, and *FmNASP* genes**.** (a) The full-length cDNA of *FmCyclinB* (b) The full-length cDNA of *FmNanos* (c) The full-length cDNA of *FmNASP*. Letters in circles are predicted phosphorylated amino acids, and sequences in boxes are predicted kinase motif sequences. Red and yellow boxes in (b) show Cys-Cys-His-Cys zinc finger motifs of Nanos.
